# ADAMTS14 Gene Polymorphism and Environmental Risk in the Development of Oral Cancer

**DOI:** 10.1371/journal.pone.0159585

**Published:** 2016-07-27

**Authors:** Shih-Chi Su, Ming-Ju Hsieh, Yu-Fan Liu, Ying-Erh Chou, Chiao-Wen Lin, Shun-Fa Yang

**Affiliations:** 1 Whole-Genome Research Core Laboratory of Human Diseases, Chang Gung Memorial Hospital, Keelung, Taiwan; 2 Department of Dermatology, Drug Hypersensitivity Clinical and Research Center, Chang Gung Memorial Hospital, Linkou, Taiwan; 3 Institute of Medicine, Chung Shan Medical University, Taichung, Taiwan; 4 Cancer Research Center, Changhua Christian Hospital, Changhua, Taiwan; 5 Graduate Institute of Biomedical Sciences, China Medical University, Taichung, Taiwan; 6 Department of Biomedical Sciences, Chung Shan Medical University, Taichung, Taiwan; 7 Department of Medical Research, Chung Shan Medical University Hospital, Taichung, Taiwan; 8 School of Medicine, Chung Shan Medical University, Taichung, Taiwan; 9 Institute of Oral Sciences, Chung Shan Medical University, Taichung, Taiwan; 10 Department of Dentistry, Chung Shan Medical University Hospital, Taichung, Taiwan; Ohio State University Medical Center, UNITED STATES

## Abstract

**Background:**

Oral cancer is a common malignancy that is shown to be causally associated with hereditary and acquired factors. ADAMTS14 is a member of the ADAMTS (a disintegrin-like and metalloproteinase domain with thrombospondin motifs) metalloproteinase family that plays an important role in extracellular matrix (ECM) assembly and degradation. Elevation or deficiency of certain ADAMTS proteinases has been known to be implicated in a wide range of pathological processes including atherosclerosis, arthritis, and cancer. The present study aimed to explore the impact of ADAMTS14 gene polymorphisms, combined with environmental risks on the susceptibility to oral tumorigenesis.

**Methodology/Principal Findings:**

Four single-nucleotide polymorphisms (SNPs) of the ADAMTS14 gene, including rs10823607, rs12774070, rs4747096, and rs61573157 were evaluated from 1200 normal controls and 850 patients with oral cancer. We failed to detect a significant association of four individual SNPs with oral cancer between case and control group. However, while considering behavioral exposure of environmental carcinogens, the presence of four ADAMTS14 SNPs, combined with betel nut chewing and/or smoking, profoundly leveraged the risk of oral cancer. Moreover, we observed a significant association of rs12774070, which is predicted to alter the expression and function of ADAMTS14 by *in silico* and bioinformatics analyses, with poor tumor cell differentiation (AOR: 0.59; 95% CI: 0.38–0.92; *p* = 0.02) in patients who chewed betel nuts.

**Conclusions:**

These results implicate the interaction between ADAMTS14 gene polymorphisms and environmental mutagens as a risk factor of oral tumorigenesis and suggest a correlation of rs12774070 with the degree of oral tumor cell differentiation.

## Introduction

Oral squamous cell carcinoma (OSCC) is a group of malignant lesions developing in the oral cavity and accounts for the vast majority (approximately 90%) of oral neoplasms [1, 2]. In spite of the current progresses in surgery and other treatment options, the fatality of oral cancer has remained mostly unchanged over the past decades [3, 4]. The occurrence has not significantly improved although the etiological studies of oral cancer have uncovered several parameters that contribute to the development of OSCC [5–8]. It is demonstrated that recognized risk factors of oral cancer mainly include human papillomavirus (HPV) infection [5] and habitual exposure of cancer-causing substances, such as tobacco and alcohol use and betel nut chewing [6, 9]. Besides, oral carcinogenesis is known to be modulated by genetic alterations that affect cell cycle, apoptosis, and DNA repair [7, 10]. Considering the heterogeneous nature and complex pathogenicity of cancer, all these factors seem to be reciprocal and needed to evaluate the disease prognosis.

ADAMTS (a disintegrin and metalloprotease with thrombospondin motifs) proteases represent an extracellular zinc metalloproteinase family with 19 members in humans. These multidomain proteases are known to catalyze a great variety of substrates in the extracellular matrices (ECM) [11]. Current studies have unveiled their association with a wide range of physiological and pathological processes including blood coagulation, atherosclerosis, wound healing, angiogenesis, fertility, arthritis and cancer [12]. ADAMTS14 is a recently discovered procollagen N-propeptidase that functions in reducing the solubility of the collagen molecules and facilitating their assembly into cylindrical collagen fibrils. In addition to its enzymatic activity, ADAMTS14, sharing a sequence and functional homology with ADAMTS2 and ADAMTS3, is characterized by the presence of four thrombospondin type I domains (TSR1) and a C-terminal procollagen N-proteinase (PNP) domain comprising a “protease and lacunin” (PLAC) domain. An anti-tumor effect of ADAMTS2 through interrupting intratumoral vascularization has been demonstrated [13]. Although at a lower level, ADAMTS14 is usually co-expressed with ADAMTS2 in all type I collagen-rich connective tissues to be responsible for ECM remodeling [14].

Genetic associations of ADAMTS14 with several conditions have been documented. Polymorphisms within the ADAMTS14 gene have been shown to influence genetic predisposition to multiple sclerosis [15]. Several other studies have implicated ADAMTS14 gene variations as a risk component in knee osteoarthritis and Achilles tendon pathology [16–18]. In recent years, there has been an explosion of reports concerning how these ADAMTS proteases can influence tumor microenvironment to potentiate cancer progression. However, little is known regarding the joint effects of ADAMTS14 gene polymorphisms and behavioral exposure of cancer-causing substances on the predisposition to oral cancer. Here, we conducted a hypothesis-driven case-control study to explore the effects of the interactions of ADAMTS14 gene polymorphisms with the environmental carcinogens on the risk of OSCC.

## Materials and Methods

### Subjects

This case-control study encompassed 850 male patients with oral squamous cell carcinoma and 1200 cancer-free male controls, with the approval by the institutional review board of Chung Shan Medical University Hospital in Taichung, Taiwan. Participants were recruited from 2008 to 2015. Among the 850 cases, tumors were located in the buccal mucosa, tongue, gingiva, palate, floor of the mouth, and others. Oral cancer patients were staged clinically at the time of diagnosis according to the TNM staging system of the American Joint Committee on Cancer (AJCC) [19]. Tumor differentiation was examined by a pathologist and rated according to the AJCC classification. Subjects with neither self-reported history of cancer of any sites nor oral precancerous disease such as oral submucous fibrosis, leukoplakia, erythroplakia, verrucous hyperplasia, etc. were recruited to the control group. All participants provided informed written consent at enrollment. Data on age, gender, alcohol drinking, betel quid chewing, and cigarette smoking were recorded from each participant. Betel quid chewing and alcohol drinking are defined as behavioral use of betel nuts (or related products) and alcoholic drinking, respectively. Cigarette smoking is defined as current smoking of at least one cigarette per day during the latest three months. The Institutional Review Board of Chung Shan Medical University Hospital approved this study (CSMUH No: CS13214-1), and informed written consent was obtained from each participant.

### Selection and genotyping of SNPs

In the present study, the selection of four common polymorphisms from *ADAMTS14* gene is based on their wide associations with the development of miscellaneous pathophysiological processes [16, 18, 20, 21]. To obtain adequate power for testing the potential association and evaluate the putative functional relevance of ADAMTS14, four non-synonymous SNPs, including rs10823607, rs12774070, rs4747096, and rs61573157, with minor allele frequencies >5% were chosen (Table 1). Genomic DNA was isolated from 3 mL of blood using QIAamp DNA blood mini kits (Qiagen, Valencia, CA, USA). Assessment of allelic discrimination for the *ADAMTS14* SNPs was performed by using the TaqMan assay with an ABI StepOne™ Real-Time PCR System (Applied Biosystems, Foster City, CA, USA), and further evaluated with SDS version 3.0 software (Applied Biosystems). The total volume of TaqMan assays was 10 μL, containing 5 μL of Master Mix, 0.25 μL of probes, and 10 ng of genomic DNA. The real-time PCR reaction included an initial denaturation step at 95°C for 10 min, followed by 40 amplification cycles, each consisting of 95°C for 15 sec and 60°C for 1 min.

**Table 1:** *ADAMTS14* gene polymorphisms assessed in this study.

Variable	Exon (contiguous position)			
Chromosome	10:70741007	10:70753879	10:70758253	10:70760503
Exon	12	19	21	22
cDNA position & nucleotide change^╪^	c.1778T>C	c.2818C>A	c.3155A>G	c.3331C>T
Protein position^†^	593	940	1052	1111
dbSNP rs No.	rs10823607	rs12774070	rs4747096	rs61573157
Function	Nonsynonymous	Nonsynonymous	Nonsynonymous	Nonsynonymous
dbSNP allele	CTG>CGG	CTG>ATG	GAA>GGA	CCA>TCA
Protein residue	L [Leu] >P [Pro]	L [Leu] >M [Met]	E [Glu]> G [Gly]	P [Pro]> S [Ser]
Codon position	2	1	2	1

╪: NM_139155.2

†: NP_631894.2.

### Statistical analysis

A chi-square goodness-of-fit test was used to evaluate Hardy-Weinberg equilibrium for biallelic markers. The differences in demographic parameters between OSCC patients and cancer-free controls were estimated by using Fisher’s exact test or parametric independent t-test. The adjusted odds ratios (AORs) with their 95% confidence intervals (CIs) for the association between genotype frequencies and the risk of OSCC plus clinicopathological characteristics were estimated by multiple logistic regression models after controlling for other covariates. The interactions of ADAMTS14 gene polymorphisms with each demographic characteristic were evaluated as described previously [22]. The association of rs12774070 genotypes with the expression levels of ADAMTS14 in esophagus mucosa tissues was assessed by one-way ANOVA. A *p* value < 0.05 was considered significant. The data were analyzed by using SAS statistical software (Version 9.1, 2005; SAS Institute Inc., Cary, NC).

### Bioinformatics analysis

Several bioinformatics tools were used to assess a putative functional relevance of rs12774070. Data from the Genotype-Tissue Expression (GTEx) database were used to identify the correlations between rs12774070 and ADAMTS14 expression in esophagus mucosa tissues [23]. The homology model of TSR1 (3) domain of ADAMTS14 was illustrated with the ribbon diagram (ViewerLite 5.0) by using Swiss Model sever (template as PDB accession number: 3GHM, 40.43% identity). Candidate deleterious non-synonymous SNPs were identified using SIFT [24] and PolyPhen-2 [25].

## Results

In this study, 850 patients with oral cancer and 1200 normal controls were recruited to explore the risk effect of ADAMTS14 gene polymorphisms (rs10823607, rs12774070, rs4747096, and rs61573157) on the development of oral neoplasm. The demographic characteristics of two study groups as well as the clinical parameters of cancer patients were evaluated (Table 2). Only male participants are enrolled to bypass the impact of gender differences on disease etiology. In accordance with our previous data [22] and findings from others [3, 4], significant differences in smoking, alcohol drinking, and betel nut chewing were observed between healthy participants and OSCC patients.

**Table 2:** The distributions of demographical characteristics in 1200 male controls and 850 male patients with oral cancer.

Variable	Controls (N = 1200)	Patients (N = 850)	*p* value
Age (yrs)	Mean ± S.D.	Mean ± S.D.	
	53.91 ± 10.02	54.60 ± 11.09	*p* = 0.14
Betel quid chewing			
No	1001 (83.4%)	156 (18.4%)	
Yes	199 (16.6%)	694 (81.6%)	*p* <0.01*
Cigarette smoking			
No	564 (47.0%)	85 (10.0%)	
Yes	636 (53.0%)	765 (90.0%)	*p* <0.01*
Alcohol drinking			
No	963 (80.3%)	357 (42.0%)	
Yes	237 (19.7%)	493 (58.0%)	*p* <0.01*
Stage			
I+II		412 (48.5%)	
III+IV		438 (51.5%)	
Tumor T status			
T1+T2		489 (57.5%)	
T3+T4		361 (42.5%)	
Lymph node status			
N0		570 (67.1%)	
N1+N2+N3		280 (32.9%)	
Metastasis			
M0		841 (98.9%)	
M1		9 (1.1%)	
Cell differentiation			
Well differentiated		141 (16.6%)	
Moderately or poorly differentiated		709 (83.4%)	

Parametric independent t-test was used between healthy controls and patients with oral cancer.

* *p* value < 0.05 as statistically significant.

To test the potential influence of ADAMTS14 gene polymorphisms on the development of OSCC, four non-synonymous single-nucleotide polymorphisms (nsSNPs), rs10823607, rs12774070, rs4747096, and rs61573157 were evaluated in this investigation. The distributions of genotype frequencies for each SNP were shown in Table 3. In these controls, the genotypic frequency of ADAMTS14 SNP rs10823607, rs12774070, rs4747096, and rs61573157 met the Hardy-Weinberg equilibrium (*p* = 0.87, χ^2^ value: 0.02; *p* = 0.10, χ^2^ value: 2.76; *p* = 0.26, χ^2^ value: 1.27; and *p* = 0.65, χ^2^ value: 0.20, respectively). Moreover, in the case group, the frequencies of four selected SNPs also met the Hardy-Weinberg equilibrium (p = 0.42, χ2 value: 0.64; p = 0.37, χ2 value: 0.79; p = 0.82, χ2 value: 0.05; and p = 0.98, χ2 value: <0.01, respectively). We failed to individually detect any significant association of these ADAMTS14 variants with the occurrence of oral cancer between two study groups either with or without the adjustment for betel quid chewing, cigarette smoking, and alcohol drinking. To further clarify the joint effects of ADAMTS14 gene variations and environmental factors on the incidence of OSCC, two common carcinogens, betel quid chewing and cigarette smoking, were chosen to examine their impacts with ADAMTS14 gene polymorphisms on the susceptibility to oral cancer. Among 1401 smokers, subjects who are betel nut users and carriers of wild-type allele of rs10823607, rs12774070, rs4747096, and rs61573157 (CC, CC, AA and CC, respectively) are more inclined to develop oral cancer than are those neither chewing betel nut nor possessing the polymorphic allele (rs10823607: AOR, 13.07; 95%CI, 9.40–18.18; rs12774070: AOR, 15.05; 95%CI, 10.43–21.70; rs4747096: AOR, 24.74; 95%CI, 14.38–42.57; and rs61573157: AOR, 16.72; 95%CI, 11.80–23.69) (Table 4). This risk is further heightened when individuals who not only chew betel nut but also carry at least one minor allele of rs10823607, rs12774070, rs4747096, and rs61573157 (rs10823607: AOR, 14.45; 95%CI, 8.38–24.93; rs12774070: AOR, 15.36; 95%CI, 9.53–24.77; rs4747096: AOR, 17.17; 95%CI, 10.55–27.94; and rs61573157: AOR, 13.90; 95%CI, 8.13–23.76). Though the genetic contribution alone appears to be subtle, a significant interaction between betel nut chewing and the existence of at least one polymorphic allele of these four ADAMTS14 SNPs is shown to be correlated with the incidence of oral cancer in smokers.

**Table 3:** Distributions of genotypic frequencies of *ADAMTS14* SNPs in controls and oral cancer patients.

Variable	Controls (N = 1200) n (%)	Patients (N = 850) n (%)	OR (95% CI)	AOR (95% CI)
**rs10823607**				
CC	1017 (84.7%)	714 (84.0%)	1.00	1.00
CT	175 (14.6%)	132 (15.5%)	1.07 (0.84–1.37)	1.03 (0.74–1.42)
TT	8 (0.7%)	4 (0.5%)	0.71 (0.21–2.37)	0.80 (0.15–4.18)
CT+TT	183 (15.3%)	136 (16.0%)	1.06 (0.83–1.35)	1.02 (0.74–1.40)
**rs12774070**				
CC	876 (73.0%)	616 (72.5%)	1.00	1.00
CA	290 (24.2%)	219 (25.8%)	1.07 (0.88–1.32)	1.06 (0.81–1.39)
AA	34 (2.8%)	15 (1.8%)	0.63 (0.34–1.16)	0.47 (0.21–1.07)
CA+AA	324 (27.0%)	234 (27.5%)	1.03 (0.84–1.25)	0.99 (0.76–1.29)
**rs4747096**				
AA	489 (40.8%)	360 (42.4%)	1.00	1.00
AG	568 (47.3%)	384 (45.2%)	0.92 (0.76–1.11)	1.00 (0.78–1.29)
GG	143 (12.0%)	106 (12.5%)	1.01 (0.76–1.34)	1.19 (0.81–1.75)
AG+GG	711 (59.3%)	490 (57.6%)	0.94 (0.78–1.12)	1.04 (0.82–1.32)
**rs61573157**				
CC	995 (82.9%)	702 (82.6%)	1.00	1.00
CT	194 (16.2%)	141 (16.6%)	1.03 (0.81–1.31)	1.03 (0.75–1.41)
TT	11 (0.9%)	7 (0.8%)	0.90 (0.35–2.34)	1.31 (0.36–4.85)
CT+TT	205 (17.1%)	148 (17.4%)	1.02 (0.81–1.29)	1.04 (0.76–1.42)

The odds ratio (OR) with their 95% confidence intervals were estimated by logistic regression models.

The adjusted odds ratio (AOR) with their 95% confidence intervals were estimated by multiple logistic regression models after controlling for betel nut chewing, alcohol and tobacco consumption.

**Table 4:** Associations of combined effects of *ADAMTS14* genotypic frequencies and betel nut chewing with oral cancer among 1401 smokers.

Variable	Controls (n = 636) (%)	Patients (n = 765) (%)	OR (95% CI)	AOR (95% CI)
**rs10823607**				
^a^ CC genotype & non-betel nut chewing	372 (58.5%)	91 (11.9%)	1.00	1.00
^b^CT or TT genotype & non-betel nut chewing	77 (12.1%)	13 (1.7%)	0.70 (0.37–1.30)	0.54 (0.26–1.13)
^c^CC genotype with betel nut chewing	160 (25.2)	556 (72.7%)	**14.21 (10.64–18.97)**	**13.07 (9.40–18.18)**
^d^CT or TT genotype with betel nut chewing	27 (4.2%)	105 (13.7%)	**15.90 (9.83–25.72)**	**14.45 (8.38–24.93)**
**rs12774070**				
^a^ CC genotype & non-betel nut chewing	326 (51.3%)	75 (9.8%)	1.00	1.00
^b^CA or AA genotype & non-betel nut chewing	123 (19.3%)	29 (3.8%)	1.03 (0.64–1.65)	0.89 (0.51–1.55)
^c^CC genotype with betel nut chewing	137 (21.5%)	478 (62.5%)	**15.17 (11.07–20.78)**	**15.05 (10.43–21.70)**
^d^CA or AA genotype with betel nut chewing	50 (7.9%)	183 (23.9%)	**15.91 (10.65–23.76)**	**15.36 (9.53–24.77)**
**rs4747096**				
^a^AA genotype & non-betel nut chewing	190 (29.9%)	36 (4.7%)	1.00	1.00
^b^AG or GG genotype & non-betel nut chewing	259 (40.7%)	68 (8.9%)	1.39 (0.89–2.16)	1.33 (0.80–2.22)
^c^AA genotype with betel nut chewing	73 (11.5%)	293 (38.3%)	**21.18 (13.66–32.86)**	**24.74 (14.38–42.57)**
^d^AG or GG genotype with betel nut chewing	114 (17.9%)	368 (48.1%)	**17.04 (11.27–25.76)**	**17.17 (10.55–27.94)**
**rs61573157**				
^a^ CC non-betel nut chewing	375 (59.0%)	79 (10.3%)	1.00	1.00
^b^CT or TT genotype & non-betel nut chewing	74 (11.6%)	25 (3.3%)	1.60 (0.96–2.68)	1.58 (0.86–2.89)
^c^CC genotype with betel nut chewing	152 (23.9%)	549 (71.8%)	**17.15 (12.68–23.19)**	**16.72 (11.80–23.69)**
^c^CT or TT genotype with betel nut chewing	35 (5.5%)	112 (14.6%)	**15.19 (9.68–23.83)**	**13.90 (8.13–23.76)**

The odds ratios (ORs) with their 95% confidence intervals were estimated by logistic regression models.

The adjusted odds ratios (AORs) with their 95% confidence intervals were estimated by multiple logistic regression models after controlling for age and alcohol consumption.

^a^ Individual with wild-type genotype but without betel nut chewing.

^b^ Individual with either at least one minor allele without betel nut chewing.

^c^ Individual with wild-type genotype and betel nut chewing.

^d^ Individual with both at least one minor allele and betel nut chewing.

**Fig 1:**
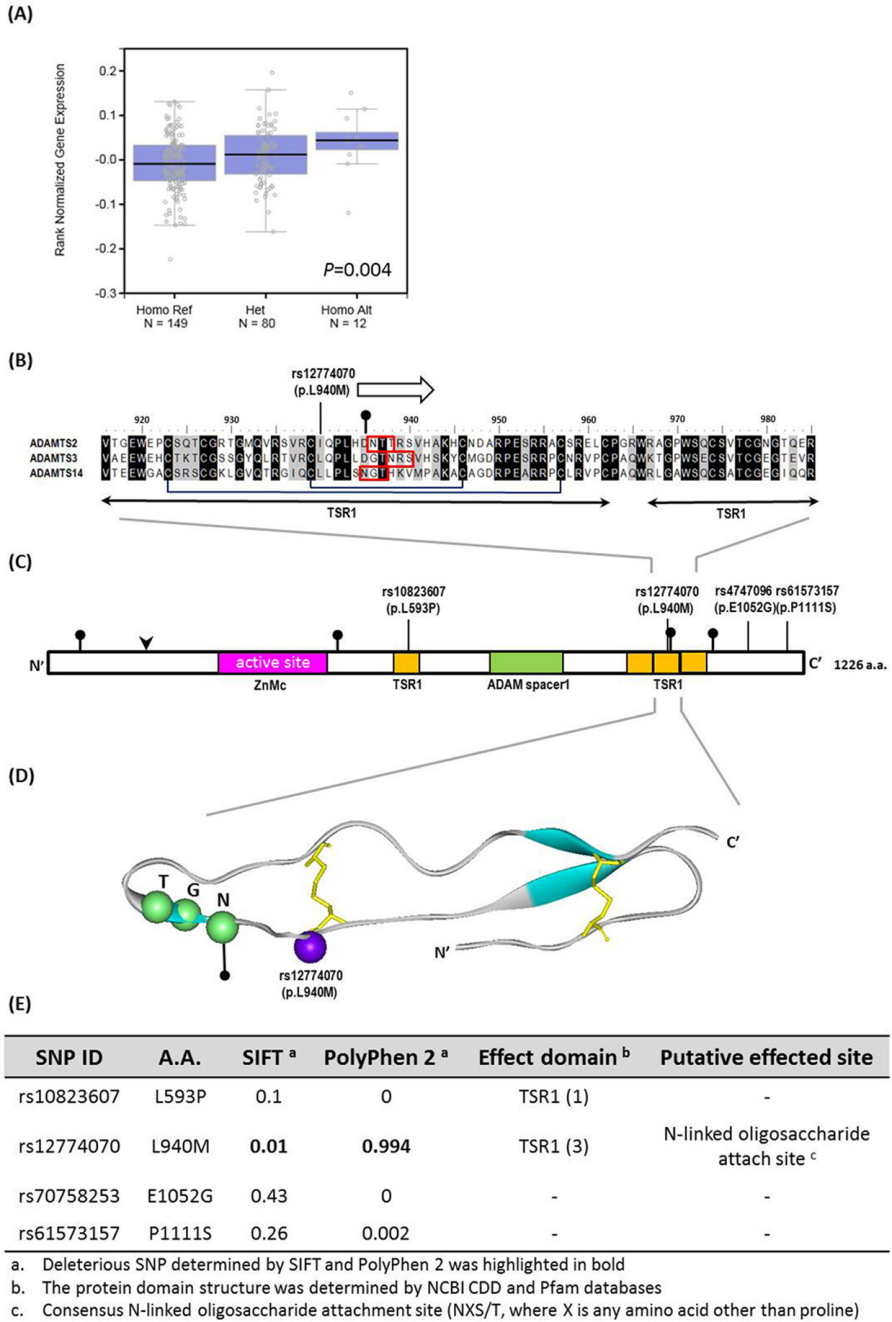
Functional implication and *In silico* profiling of ADAMTS14 SNP rs12774070. **(A)** ADAMTS14 displays a significant eQTL association with rs12774070 genotypes in esophagus mucosa tissues (GTEx data set). (**B**) The altered residue is conserved throughout three procollagen aminopropeptidase subfamily of ADAMTS proteases, including ADAMTS2 (NP_055059.2), ADAMTS3 (NP_055058.2) and ADAMTS14 (NP_631894.2), as shown by alignment of the protein sequences with Crustal Omega software. Numbering is for human ADMDTS14. The arrow represents b-strands, and consensus sites for N-liked oligosaccharide attachment are marked in red (pinpointed). The disulfide bonds are marked in dark lines connecting paired cysteines. (**C**) Schematic representation of the full-length human ADMDTS14 protein, domain symbols are drawn approximately to scale. The rectangles represent the key domain structures, ZnMc superfamily (cd04273; pink), ADAM spacer1 (pfam05986; green) and four TSR1 (smart00209; yellow) by NCBI CDD server. Putative furin cleavage site is indicated by an arrowhead, and N-linked glycosylation sites are pointed by pins. (**D**) Ribbon diagram depicts the homology model of TSR1 (3) domain of ADAMTS14. The ribbon indicates the Cα carbon of the TSR1 domain characterized in this study. The blue ribbon, purple sphere, green spheres and yellow sticks indicate the b-strands structure, SNP rs12774070, potential N-linked glycosylation site and disulfide bonds, respectively. (**E**) Pathogenicity prediction of ADAMTS14 SNPs assessed in this study.

We further analyzed the combined effect of ADAMTS14 gene polymorphisms and environmental factors on influencing the clinical status of OSCC. For 694 patients who chewed betel quid, a significant association of rs12774070 variants (CA+AA vs CC) with poor tumor cell differentiation (AOR: 0.62; 95% CI: 0.41–0.94; *p* = 0.02) but not clinical stage, tumor size, and lymph node metastasis was observed (Table 5). However, we failed to detect any significant association of the other 3 ADAMTS14 variants individually with the clinical status of OSCC in betel nut users (Data not shown). Moreover, as a preliminary assessment of the putative functional relevance of ADAMTS14 rs12774070, alteration in ADAMTS14 expression was seen in esophagus mucosa tissues of individuals who carry polymorphic allele of ADAMTS14 rs12774070 in the Genotype-Tissue Expression (GTEx) database (Fig 1A). The change in protein sequence due to the polymorphic allele of ADAMTS14 rs12774070 resides in a region of TSR1 domain, which is conserved among procollagen N-propeptidases (Fig 1B–1D). Furthermore, computational algorithms that are widely used for the discrimination of damaging nsSNPs from neutral ones have predicted ADAMTS14 rs12774070 to be deleterious (Fig 1E). There data suggest that changes in ADAMTS14 expression and function due to genetic polymorphisms, in combination with betel quid chewing, may affect tumor cell differentiation of oral cancer.

**Table 5:** Associations of genotype frequencies of *ADAMTS14* rs12774070 with clinical status of oral cancer among 694 betel quid users.

Variable	*ADAMTS14* rs12774070 (betel quid users)			
	CC (n = 503) n (%)	CA+AA (n = 191) n (%)	OR (95% CI) *p* value	AOR (95% CI) *p* value
**Clinical Stage**				
Stage I/II	248 (49.3%)	91 (47.6%)	1.00	1.00
Stage III/IV	255 (50.7%)	100 (52.4%)	1.07 (0.77–1.49) *p* = 0.70	1.10 (0.77–1.58) *p* = 0.60
**Tumor size**				
≤T2	288 (57.3%)	109 (57.1%)	1.00	1.00
> T2	215 (42.7%)	82 (42.9%)	1.01 (0.72–1.41) *p* = 0.96	1.07 (0.75–1.54) *p* = 0.71
**Lymph node metastasis**				
No	339 (67.4%)	127 (66.5%)	1.00	1.00
Yes	164 (32.6%)	64 (33.5%)	1.04 (0.73–1.45) *p* = 0.82	1.08 (0.73–1.56) *p* = 0.72
**Cell differentiation**				
well	79 (15.7%)	44 (23.0%)	1.00	1.00
Moderate/poor	424 (84.3%)	147 (77.0%)	0.62 (0.41–0.94) *p* = 0.02*	0.59 (0.38–0.92) *p* = 0.02*

The adjusted odds ratios (AORs) with their 95% confidence intervals were estimated by multiple logistic regression models after controlling for age, alcohol and tobacco consumption.

*p<0.05.

## Discussion

Converging lines of evidence has supported that development of OSCC is a complicated process regulated by both environmental and genetic factors. In the present study, we for the first time revealed that the combination of the ADAMTS14 gene polymorphisms with the behavioral use of betel nut and tobacco conferred a higher susceptibility to oral cancer. Strikingly, betel nut users bearing the polymorphic allele of ADAMTS14 rs12774070 were shown to be less likely to develop tumors with moderate/poor differentiation.

The ADAMTS family has been examined intensively for its proteolytic function on tumor angiogenesis, an indispensable process vital for cancer progression and metastasis [26]. Paradoxically, the activation of ADAMTS proteinases can exhibit both inhibitory and promotive effects on angiogenesis as the mechanism involved in their regulation of cancer development varies among different members [27]. Some members (ADAMTS2, 4, 6, and 14) are shown to be manifestly upregulated in cancer tissues, whereas others (ADAMTS1, 5, 8, 9, 10, 13, 15, and 18) are downregulated [28, 29]. In the present study, we showed a significant interaction between betel nut chewing and the presence of at least one polymorphic allele of four ADAMTS14 SNPs examined is associated with higher incidence of oral cancer in smokers. In addition, a significant increase in ADAMTS14 expression was observed in esophagus mucosa tissues of subjects who possess the polymorphic allele of ADAMTS14 rs12774070 (Fig 1A). Unlike the ADAMs (a disintegrin and metalloproteinases), the ADAMTS proteinases are secreted by the cells and are not membrane-bound. Since oral cancer is a type of malignancies that develops in the mucosa tissues of the mouth or throat, it is conceivable that the accumulation of carcinogenic substances derived from addictive behaviors (e.g. betel nut chewing and smoking) over time in combination with the elevation of specific regulators of tumor angiogenesis, such as ADAMTS14, within oral or adjacent tissues could finally lead to a malignant phenotype.

More intriguingly, we identified an inverse association of ADAMTS14 rs12774070 with cancer cell differentiation in the present study. Due to a relatively small sample size, further validating this observation will require additional experimental evidence in future investigations. The degree of epithelial cell differentiation in oral cancer is determined by the level of keratinization, a process that hardened cells filled with structurally and functionally distinct keratin [30]. A lack of cell differentiation in OSCC has been associated with more rapid tumor growth and spread, while specific keratin expression patterns are used as an independent prognostic marker and indicate a decreased overall and progression-free survival [31]. Several factors, such as components of ECM, growth factors (e.g. epidermal growth factor, transforming growth factor alpha and beta), retinoids and calcium, are known to regulate epithelial differentiation [30, 32]. ADAMTS proteinases catalyze a great variety of ECM molecules, and the ECM also controls the activity and presentation of a wide range of growth factors [33]. Therefore, remodeling of the ECM by ADAMTSs has profound effects on cell differentiation and the consequent clinical manifestations of oral cancer. Moreover, rs12774070-derived alteration in protein sequence resides in a conserved region of TSR1(3) domain and adjacent to a bisulfide bridge and N-linked glycosylation site (Fig 1). Predictions from sequence- and structure-based algorithms show that the presence of polymorphic allele of ADAMTS14 rs12774070, rather than rs10823607, rs4747096, and rs61573157, confers a determinant impact on ADAMTS14 function. These data indicate that altered ADAMTS14 function due to genetic polymorphisms, in combination with betel quid chewing, may affect tumor cell differentiation of oral cancer by virtue of disturbance of ECM remodeling.

We presented an impact of AMAMTS14 gene variations in combination with acquired factors on the incidence and tumor cell differentiation of OSCC; however, there are several limitations in the study. One is that all habitual exposures of carcinogens were dichotomized. The effects of environmental risks on the predisposition to oral cancer may be underestimtated because of a lack of quantitative definition for betel nut chewing, drinking, and smoking. The other concern is that esophageal mucosa was selected based on anatomical relevance for evaluation from GTEx database because oral tissue is unavailable. Also unavailable are patients’ survival data since a majority of patients were enrolled in the past few years. Another weakness is that the control group in this hospital-based study was recruited among individuals without cancer. We could not entirely exclude the possibility of potential selection and recall bias. In addition, the results presented in this study may not be able to be extended to other populations unless replication studies are conducted. Furthermore, the functional role of ADAMTS14 in cell differentiation of oral cancer requires further investigation.

In conclusion, our results demonstrate that a joint effect of ADAMTS14 SNPs (rs10823607, rs12774070, rs4747096, and rs61573157) with betel nut chewing and smoking causally contributes to the occurrence of oral cancer. In addition, an inverse association of SNP ADAMTS14 rs12774070 with tumors cell differentiation was observed in betel nut users. These findings reveal a novel genetic-environmental predisposition to oral tumorigenesis.
